# Comprehensive evaluation of carbon sequestration potential of landscape tree species and its influencing factors analysis: implications for urban green space management

**DOI:** 10.1186/s13021-023-00238-w

**Published:** 2023-09-05

**Authors:** Shanshan Jin, Ershan Zhang, Haotian Guo, Chuanwei Hu, Yaru Zhang, Dongfeng Yan

**Affiliations:** 1 College of Forestry, Henan Agricultural University, No. 63, Nongye Road, Jinshui District, Zhengzhou, 450002 China; 2 Plam Eco-Town Development CO., LTD, Zhengzhou, 450002 China; 3 College of Forestry, Northwest A&F University, Yangling, 712100 China

**Keywords:** Landscape tree species, Carbon sequestration potential, Urban green space, Net photosynthetic rate

## Abstract

**Background:**

Continuous increasing carbon dioxide (CO_2_) has aggravated global warming and promoted urban tree planting projects for many countries. So it’s imperative to select high carbon sequestering landscape tree species while considering their aesthetic values of urban green space.

**Results:**

32 tree species were selected as test objects which were commonly used in landscaping in Zhengzhou, a typical northern city of China. To assess the comprehensive carbon sequestration potential of landscape tree species in different plant configuration types, we simultaneously considered their daily net carbon sequestration per unit leaf area (*wCO*_*2*_), daily net carbon sequestration per unit land area (*WCO*_*2*_) and daily net carbon sequestration of the whole plant (*QCO*_*2*_) through cluster analysis. Besides that, we found out the key factors affecting carbon sequestration potential of landscape tree species by redundancy analysis.

**Conclusion:**

*Populus*, *P Stenoptera*, *P. acerifolia* among large arbors (LA), *V odoratissimum*, *P. Serratifolia*, *S. oblata* among small arbors (SA), and *B sinica var. Parvifolia*, *B. Megistophylla*, *L quihoui* among shrubs (S) were recommended for local urban green space management. Photosynthetic rate (*Pn*), crown area (*CA*) and leaf area index (*LAI*) were the key factors which affected the comprehensive carbon sequestration potential both for LA, SA and S.

## Background

A series of ecological problems mainly caused by CO_2_ and other greenhouse gases have attracted human’s much attentions, such as glacier melting, land desertification, climate anomaly and biodiversity change [1–4]. Countries around the world successively proposed relevant plant projects to mitigate CO_2_, like Japan’s “Action Plan for Achieving A Low-carbon Society”, EU’s “Fits for 55” plan, or China’s “Carbon Peak, Carbon Neutralization” strategy [5–7]. Urban areas have become the foci of policies for mitigation actions because of the high CO_2_ emissions [8, 9], especially in China, which was facing rapid industrialization and urbanization trends as the biggest developing country in the world. Trees can reduce atmospheric CO_2_ mixing ratios by converting it into carbohydrates through photosynthesis and assimilated it into plant biomass [10–12]. For that, green space in urban and peri urban areas played a pivotal role in mitigating atmospheric CO_2_ in these settings [13, 14]. However, these results above were many based on limited scientific evidence and lack of better knowledge about quantitative study on carbon sequestration potential of landscape tree species [15, 16].

Previous studies have shown that green space in and around urban settings can contribute to ecosystem services and affect the sustainability of urban ecosystem as well as the livability of urban dwellers [17, 18], so city authorities usually tried to increase the green space areas in urban greening practice [19, 20]. However, higher carbon sequestration potential tree species may be required to achieve the carbon reduction goal when there was a limited urban space rather than increase wide-scale urban green areas. Although many researches have been carried out to discuss various plants carbon sequestration capacity, these were concentrated in the field of forest ecosystem, including of forest carbon sequestration’s evaluation, forest carbon storage’s quantifying methods, or their associated factors analysis [21–26], while rare researches involved garden plants. So it was necessary to assess the carbon sequestration potential of different landscape tree species in order to maximize their carbon reduction function [19, 27]. Besides that, most of the limited relevant studies were only on a simple assessment among some plants [28–30], while a systematic quantitative comparison for multifarious commonly used landscape tree species were very few [31, 32]. Moreover, arbors, shrubs and grasses were generally jointly used to form a hierarchical landscape space in plant configuration so as to form a balanced and sustainable pattern [33, 34]. Therefore, quantitative evaluation was needed to screen out tree species with high carbon sequestration potential, and thus providing a basis for the allocation of landscape plants.

Various methods have been used to quantify the amount of plants carbon sequestration capacity, such as biomass method, assimilation method, eddy covariance method, model simulation method and remote sensing method, etc. [35–37]. The first two were usually well received for researchers because they only required simple field investigation and convenient calculation process. In fact, the essence of biomass method was to calculate plant’s carbon storage through trees allometric growth equation and the corresponding carbon content [38, 39], or obtained by biomass expansion factor and trees basic wood density based on plant growth indexes [40, 41]. However, this method may exit some practical limitations for some countries, because the majority available tree allometry database and relevant tools like ‘i-tree’ were established in the US or the EU countries [42–44], and few developed in other countries, like China. Hence, we generally referred to relevant forest standard or used foreign trees allometric equation to assess the carbon sequestration potential of landscape tree species. Compared with biomass method, assimilation method may get a more accurate results in theory [29, 45], since carbon sequestered was the difference between carbon benefited by photosynthesis and carbon lost by respiration [46]. As evident, trees with higher carbon storage and sequestration rates also have higher net photosynthetic capacities [16]. In view of this, we used it to quantify the amount of plants carbon sequestration from different aspects.

Zhengzhou City is located in northern China with warm temperate continental monsoon climate, and the common landscape tree species here have the typical characteristics of landscape vegetation configuration in this climatic zone [47]. There are more than 200 species of landscape tree species in Zhengzhou now. However, few quantitative studies are devoted to analyzing the carbon sequestration potential of landscape tree species in this or similar areas nearby. Therefore, we investigated and assessed 32 commonly used landscape tree species with dominant quantity and planting area in urban green space based on local actual situation.

The main goals of this study are as follows: Firstly, evaluate the comprehensive carbon sequestration potential of 32 common landscape tree species; Secondly, assess the carbon sequestration potential of different plant configuration types; Thirdly, find out the key factors that affect carbon sequestration potential of landscape tree species. Our results are expected to provide a scientific basis for rational allocation of landscape tree species and quantitative evaluation of environmental benefits.

## Methods

### Study area

Zhengzhou (112°42′-114°14′E, 34°16′-34°58′N) is located in the north–south transition zone of China, which has rich tree species [47]. It is also at the boundary between the middle and lower reaches of the Yellow River. Total area is 7567 km^2^, of which the built-up area of central urban area is 744.15 km^2^, and the urbanization rate is as high as 79.1%. Terrain is generally high in the southwest and low in the northeast. Also, it is characterized by four distinct seasons with temperate continental monsoon climate. Average annual temperature here is 14.7°, with the lowest temperature in January and the highest temperature in July. Besides, average annual rainfall is 632.4 mm, mainly from June to August, and average annual frost free period is 212.6 days. Soil type in this area belongs to brown soil and cinnamon soil, zonal vegetation belongs to temperate deciduous evergreen mixed broad-leaved forest belt, and flora belongs to north central temperate distribution and east Asia distribution.

### Selection of garden plants

32 common landscape tree species were selected in this test through a comprehensive survey in the urban area of Zhengzhou, including of 17 large arbors (9 deciduous large arbors, 8 evergreen large arbors), 8 small arbors (5 deciduous small arbors, 3 evergreen small arbors) and 7 shrubs (4 deciduous shrubs and 3 evergreen shrubs) (Table 1). Tested tree species were investigated in the Wenhua Road Campus of Henan Agricultural University in central Zhengzhou, which had a green area of 6.67 × 10^4^ m^2^. All trees grew at a relatively consistent climatic environment and management conditions. Also, the age of all trees was 10–15 years, and they were all healthy plants without obvious diseases and pests. Our study was carried out in the sunny weather which had sufficient natural light source without wind and rain from July to September in 2022 when plants grew vigorously. The average air temperature, relative humidity, duration of sunlight, wind speed and total solar radiation was 27.0 ℃, 68.2%, 5.3 h d^−1^, 9.5 km h^−1^ and 18185.7 kJ m^−2^ d^−1^, respectively, and the total rainfall was 224.2 mm during the investigation period.

**Table 1:** Test Plant Materials

Number	Tree species	Plant configuration types	Family
1	*Acer buergerianum*	Deciduous large arbors (DLA)	Aceraceae
2	*Koelreuteria paniculata*		Sapindaceae
3	*Ginkgo biloba* L		Ginkgoaceae
4	*Platanus acerifolia* (Aiton) Willdenow		Campanulaceae
5	*Salix babylonica* L		Salicaceae
6	*Pterocarya stenoptera* C. DC		Juglandaceae
7	*Styphnolobium japonicum* (L.) Schott		Leguminosae
8	*Populus* L		Salicaceae
9	*Yulania denudata (Desr.) D. L. Fu*		Magnoliaceae
10	*Eriobotrya japonica* (Thunb.) Lindl	Evergreen large arbors (ELA)	Rosaceae
11	*Cinnamomum camphora (L.) presl*		Lauraceae
12	*Ligustrum compactum* (Wall. ex G. Don) Hook. f		Oleaceae
13	*Pinus bungeana* Zucc		Pinaceae
14	*Platycladus orientalis* (L.) Franco		Cupressaceae
15	*Sabina chinensis* (L.) Ant. cv. Kaizuca		Cupressaceae
16	*Juniperus formosana* Hayata		Cupressaceae
17	*Magnolia grandiflora* L		Magnoliaceae
18	*Amygdalus persica ‘Duplex’*	Deciduous small arbors (DSA)	Rosaceae
19	*Syringa oblata* Lindl		Oleaceae
20	*Cercis chinensis* Bunge		Leguminosae
21	*Prunus subg. Cerasys sp.*		Rosaceae
22	*Prunus cerasifera ‘Atropurpurea’*		Rosaceae
23	*Viburnum odoratissimum* Ker.-Gawl	Evergreen small arbors (ESA)	Caprifoliaceae
24	*Osmanthus fragrans* (Thunb.) Lour		Oleaceae
25	*Photinia serratifolia* (Desfontaines) Kalkman		Rosaceae
26	*Punica granatum* L	Deciduous shrubs (DS)	Pomegranaceae
27	*Chimonanthus praecox* (L.) Link		Chimonaceae
28	*Ligustrum quihoui* Carr		Oleaceae
29	*Amygdalus triloba*		Rosaceae
30	*Buxus megistophylla* Levl	Evergreen shrubs (ES)	Buxaceae
31	*Pittosporum glabratum* Lindl		Pittosporaceae
32	*Buxus sinica var. parvifolia* M. Cheng		Buxaceae

### Determination of influence indexes

Photosynthetic indexes, growth indexes, physiological indexes and Leaf area index (*LAI*) were selected in our studies for their potential relationship with trees carbon sequestration capacity according to previous studies [46, 48–50].

Determination of photosynthetic indexes: The LCpro SD portable photosynthetic instrument (made by ADC BioScientific Ltd., in the UK) was used to measure the photosynthetic physiological and ecological indicators of the tested tree species. Three trees with similar growth vigor were selected for all tested tree species, and five undamaged, well grown and mature leaves were selected for each tree for determination. The sampling work started at 8:00 and ended at 18:00, and the measurement was conducted every 2 h. Measurement work started when the instrument system was stable. Six instantaneous photosynthetic rate (*Pn*) values were recorded for each leaf, and the average value was finally taken. Meanwhile, transpiration rate (*Er*), stomatal conductance (*Ci*), intercellular CO_2_ concentration (*Gs*), etc., were recorded.

Determination of growth indexes: girth was used to measure the diameter at breast height (*DBH*) of all tested trees, and laser rangefinder was used to measure their height (*H*) and crown diameter (*CD*). Besides, crown area (*CA*) was calculated by estimation method, which was the result of product of the east–west and north–south crown diameter.

Determination of physiological indexes: Chlorophyll content (*CHl*): select 15 mature and fully developed leaves, wipe the surface of the leaves with a paper towel and waite for measurement. Used a portable chlorophyll meter (SPAD-502 PLUS) to measure the chlorophyll content of the leaves. Avoided the veins and petioles when measuring, and finally taken the average value.

Leaf area index (*LAI*): take canopy photos with a digital camera which connected to a fisheye lens at a height of 1.65 m above the tested tree species, and use Gap Light Analyzer (GLA) Version 2.0 to identify and analyze the clear photos that were easy to distinguish the sky and the canopy, and then get the leaf area index after sorting.

### Calculation of test indexes

Photosynthetic carbon sequestration index: based on Han Huanjin’s calculation principle of daily assimilation amount of photosynthesis, the net assimilation amount of plants on the day of measurement was used to estimate the amount of carbon sequestration of plants [52].

1 $$p = \sum\nolimits_{(i = 1)}^{n} {\frac{{(p_{(i + 1)} + p_{i} ) \times (t_{(i + 1)} - t_{i} )}}{2 \times 1000}} \times 3600$$

2 $$q = \frac{{\mathop \sum \nolimits_{i = 1}^{n} p_{i} }}{n}$$

where, *p* is the total daily net assimilation amount per unit leaf area of tree species(mmol m^−2^ d^−1^), and* p*_*i*_ is the instantaneous cooperative utilization rate at initial measurement point of tree species (μmol m^−2^ s^−1^), *p*_*i*+*1*_ is the instantaneous cooperative utilization rate of tree species at i + 1 measuring point(μmol m^−2^ s^−1^), *t*_*i*_ is the instantaneous time (h) of the initial measuring point of the tree species, *t*_*i*+*1*_ is the instantaneous time (h) of the tree species at the measuring point i + 1, *n* is the number of tests, *q* is the daily average photosynthetic rate (μmol m^−2^ s^−1^), 3600 represents 3600 s per hour, and 1000 represents 1000 μmol per 1 mmol.

The amount of plants carbon sequestration was calculated according to the reaction equation of photosynthesis: CO_2_ + 4H_2_O → CH_2_O + 3H_2_O + O_2_. And daily net carbon sequestration amount was converted by daily net assimilation amount.

Daily net carbon sequestration per unit leaf area (*wCO*_*2*_):

3 $$\omega_{{co_{2} }} = \frac{p \times 44}{{1000}} \times \left( {1 - 20\% } \right)$$

Daily net carbon sequestration per unit land area (*WCO*_*2*_):

4 $$W_{{CO_{2} }} = \omega_{{CO_{2} }} \times LAI$$

Daily net carbon sequestration of the whole plant (*QCO*_*2*_):

5 $$Q_{{CO_{2} }} = W_{{CO_{2} }} \times CA$$

where, ${\omega }_{{co}_{2}}$ is the daily net carbon sequestration per unit leaf area of tree species(gm^−2^ d^−1^). The night respiration consumption of the tested tree species was calculated as 20% of the total net assimilation amount of the tree species in the day [53]. 44 is the molar mass of CO_2_. ${W}_{C{O}_{2}}$ is the daily net carbon sequestration per unit land area of tree species(gm^−2^ d^−1^), ${Q}_{C{O}_{2}}$ is the daily net carbon sequestration of the whole plant (g.d^−1^)*, LAI* is the leaf area index, and *CA* is the crown area (m^2^).

Coefficient of variation (*CV*)

6 $$CV = {\raise0.7ex\hbox{$s$} \!\mathord{\left/ {\vphantom {s {\overline{x}}}}\right.\kern-0pt} \!\lower0.7ex\hbox{${\overline{x}}$}}$$

where, *CV* is the coefficient of variation. According to Wilding’s classification of variation degree, it indicates weak variation when *CV* ≤ 15%, and indicates medium variation when 16 ≤ *CV* ≤ 35%, and indicates strong variation when *CV* ≥ 36% [55].

### Statistics and analysis

In this study, R 4.1.3 (R Core Team, Vienna, Austria), Canoco 5 (Microcomputer Power, NY, USA) and Origin 2021 (OriginLab, Northampton, MA, USA) were used for all statistical analyses. The comprehensive carbon sequestration potential was assessed with cluster analysis method by R packages “cluster” [56]. And the variance and cluster analysis was conducted by using the R packages “multcomp” [57]. Besides, the key factors which affected trees carbon sequestration potential were analyzed with redundancy analysis method by Canoco 5.

## Results

### Description of basic characteristics of landscape tree species

Average value of four morphological indicators of landscape tree species in six plant configuration types were displayed in Table 2. It can be seen that there were obvious differences in *DBH*, *H* and *CA* for all types, which were generally shown as LA (large arbors) > SA (small arbors) > S (shrubs). In addition, *DBH*, *H* and *CA* of each tree species in a same plant configuration types also differed considerably. Specifically, *DBH* (34.3% < *CV* < 60.2%) and *H* (25.5% < *CV* < 42.9%) both showed moderate or strong variability, but except for deciduous small arbors (DSA, *CV* = 8.1%) and evergreen small arbors (ESA, *CV* = 3.9%), respectively, and *CA* in all types showed strong variability (40.7% < *CV* < 92.3%). However, the value of *LAI* was close to 2.40 for all types, and had less variation than other morphological indicators, which showed weak or medium variation. Overall, it varied greatly for morphological characteristics among different types or different tree species in one plant configuration type by comprehensively considering *DBH*, *H*, *CA* and *LAI*.

**Table 2:** Morphological characteristics of different plant configuration types

Plant configuration types		^*13*^*DBH*(cm)		^*14*^*H*(m)		^*15*^*CA*(m^2^)		^*16*^*LAI*	
		*M*^4^ ± *SD*^*5*^	*CV*^*6*^	*M* ± *SD*	*CV*	*M* ± *SD*	*CV*	*M* ± *SD*	*CV*
LA^1^	DLA^7^	32.75 ± 11.23	34.3%	11.33 ± 2.95	26.1%	52.9 ± 28.0	53.0%	2.34 ± 0.43	18.4%
	ELA^8^	25.54 ± 9.50	37.2%	7.95 ± 2.36	29.7%	41.5 ± 28.2	68.0%	2.15 ± 0.23	10.8%
SA^2^	DSA^9^	11.15 ± 0.90	8.1%	4.61 ± 1.18	25.5%	14.4 ± 10.2	71.0%	2.43 ± 0.29	12.0%
	ESA^10^	11.71 ± 4.78	40.8%	5.37 ± 0.21	3.9%	19.1 ± 9.2	48.3%	2.55 ± 0.29	11.5%
S^3^	DS^11^	9.72 ± 4.87	50.1%	3.95 ± 1.30	32.9%	15.9 ± 6.5	40.7%	2.51 ± 0.41	16.3%
	ES^12^	10.05 ± 6.05	60.2%	2.50 ± 1.07	42.9%	17.4 ± 16.0	92.3%	2.42 ± 0.70	26.9%

^1^LA, ^2^SA and ^3^S represent ‘large arbors’, ‘small arbors’ and ‘shrubs’, respectively; ^4^* M,* ^5^*SD* and ^*6*^*CV* represent ‘mean’, ‘standard deviation’ and ‘coefficient of variation’, respectively; ^7^DLA, ^8^ELA, ^9^DSA, ^10^ESA, ^11^DS, ^12^ES represent ‘deciduous large arbor’, ‘evergreen large arbor’, ‘deciduous small arbor’, ‘evergreen small arbor’, ‘deciduous shrub’, and ‘evergreen shrub’, respectively; *DBH*^13^, *H*^14^, *CA*^15^ and *LAI*^16^ represent ‘diameter at the breast height’, ‘height’, ‘crown area’ and ‘leaf area index’, respectively

Photosynthetic characteristics evaluated by *CHI*, *Pn*, *Er*, *Ci* and *Gs* showed different regularities among six plant configuration types or tree species for a same type (Table 3). The differences for *CHI* and *Ci* were small among all types, while were large for *Pn*, *Er*, *Gs*. Specifically, *Pn* and *Gs* were shown as DS > DSA > DLA > ES > ESA > ELA, while *Er* was shown as DSA > DS > DLA > ESA > ES > ELA. It can also be seen that photosynthetic capacity varied largely among tree species according to *CV* values of each indicator. Besides, photosynthetic capacity of deciduous trees presented higher than that of evergreen trees, and that of SA and S presented higher than that of LA.

**Table 3:** Photosynthetic characteristics of different plant configuration types

Plant configuration types		*CHI*^*13*^ (SPAD)		*Pn*^*14*^ (μmol m^−2^ s^−1^)		*Er*^*15*^ (μmol m^−2^ s^−1^)		*Ci*^*16*^ (μmol mol^−1^)		*Gs*^*17*^ (μmol m^−2^ s^−1^)	
		*M*^*4*^ ± *SD*^*5*^	*CV*^*6*^	*M* ± *SD*	*CV*	*M* ± *SD*	*CV*	*M* ± *SD*	*CV*	*M* ± *SD*	*CV*
LA^1^	DLA^7^	40.39 ± 3.60	8.9%	3.80 ± 0.70	18.5%	2.55 ± 0.73	28.6%	306.45 ± 32.84	10.7%	0.09 ± 0.03	34.4%
	ELA^8^	38.01 ± 18.11	47.6%	2.99 ± 0.86	28.9%	1.31 ± 0.73	55.8%	270.49 ± 34.11	12.6%	0.03 ± 0.02	58.9%
SA^2^	DSA^9^	38.28 ± 4.25	11.1%	5.26 ± 1.66	31.6%	3.45 ± 0.90	26.0%	296.53 ± 9.40	3.2%	0.13 ± 0.02	17.6%
	ESA^10^	58.79 ± 6.34	10.8%	3.17 ± 1.05	23.4%	1.95 ± 1.21	40.6%	323.38 ± 63.88	22.8%	0.05 ± 0.06	17.1%
S^3^	DS^11^	46.04 ± 3.94	8.6%	5.52 ± 3.21	58.1%	3.38 ± 1.49	44.2%	265.42 ± 13.19	5.0%	0.15 ± 0.05	31.5%
	ES^12^	54.04 ± 11.30	20.9%	3.53 ± 0.95	26.9%	1.89 ± 0.48	25.1%	250.51 ± 5.11	2.0%	0.08 ± 0.03	45.1%

The representation of ^1^LA, ^2^SA, ^3^S, ^4^* M*, ^5^*SD*, ^6^*CV*, ^7^DLA, ^8^ELA, ^9^DSA, ^10^ESA, ^11^DS and ^12^ES is the same as Table 2; ^13^*CHI,* ^14^*Pn,* ^15^*Er,* ^16^* Ci and* ^17^*Gs* represent ‘Chlorophyll content’, ‘photosynthetic rate’, ‘transpiration rate’, ‘stomatal conductance’ and ‘intercellular CO_2_ concentration’, respectively

### Carbon sequestration potential of different plant configuration types

**Fig. 1:**
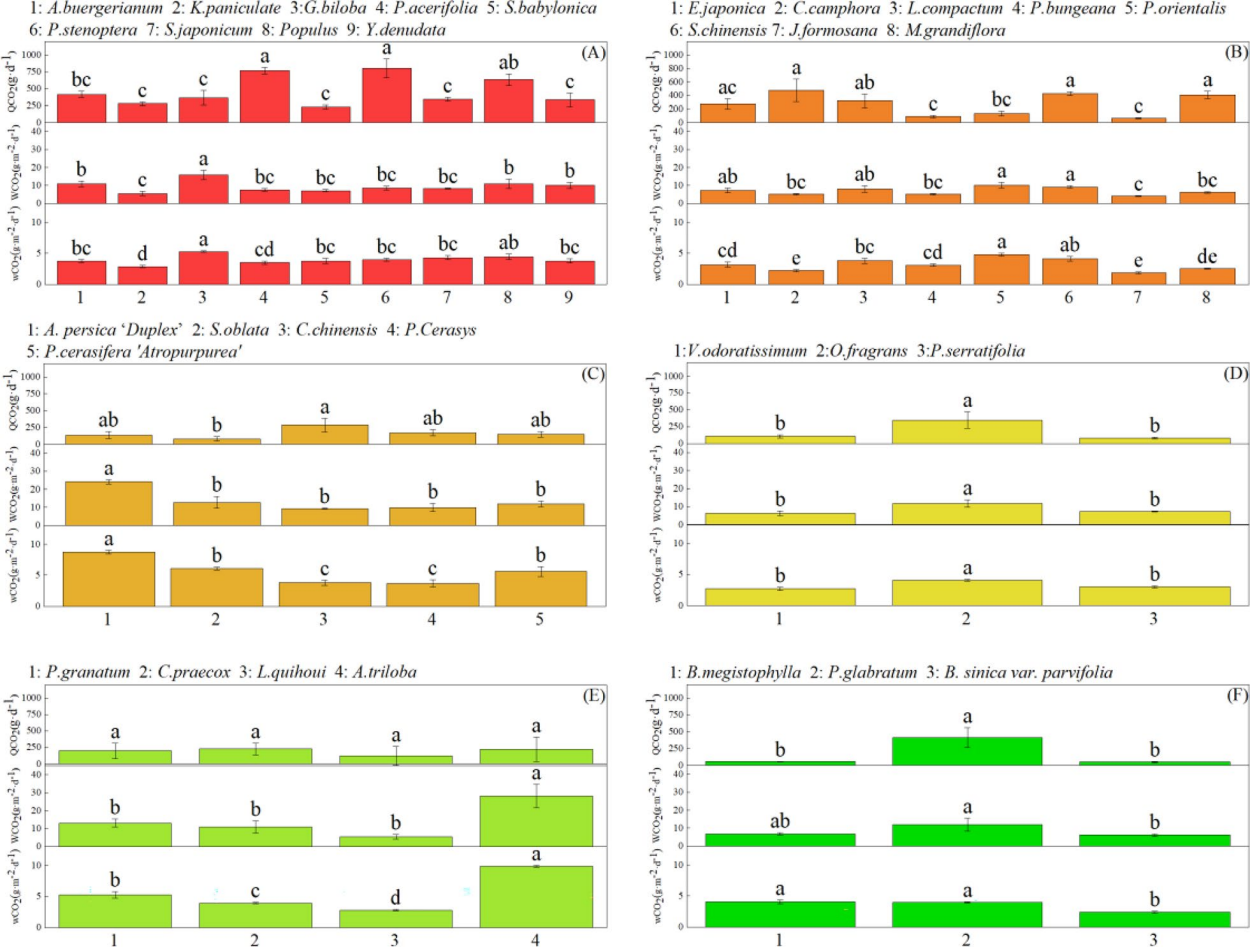
*wCO*_*2*_, *WCO*_*2*_, QCO_2_ of different tree species in different plant configuration types. A, B, C, D, E, F represent the amount of carbon sequestration (*wCO*_*2*_, *WCO*_*2*_, *QCO*_*2*_) among different tree species in DLA, ELA, DSA, ESA, DS and ES, respectively. The same letter in the figure indicates it has no significant difference between two tree species, or vice versa (*P* < *0.05*). All numbers in the horizontal axis in Fig. 1 represent one tree specie, and the names of all tree species are marked above each figure

**Fig. 2:**
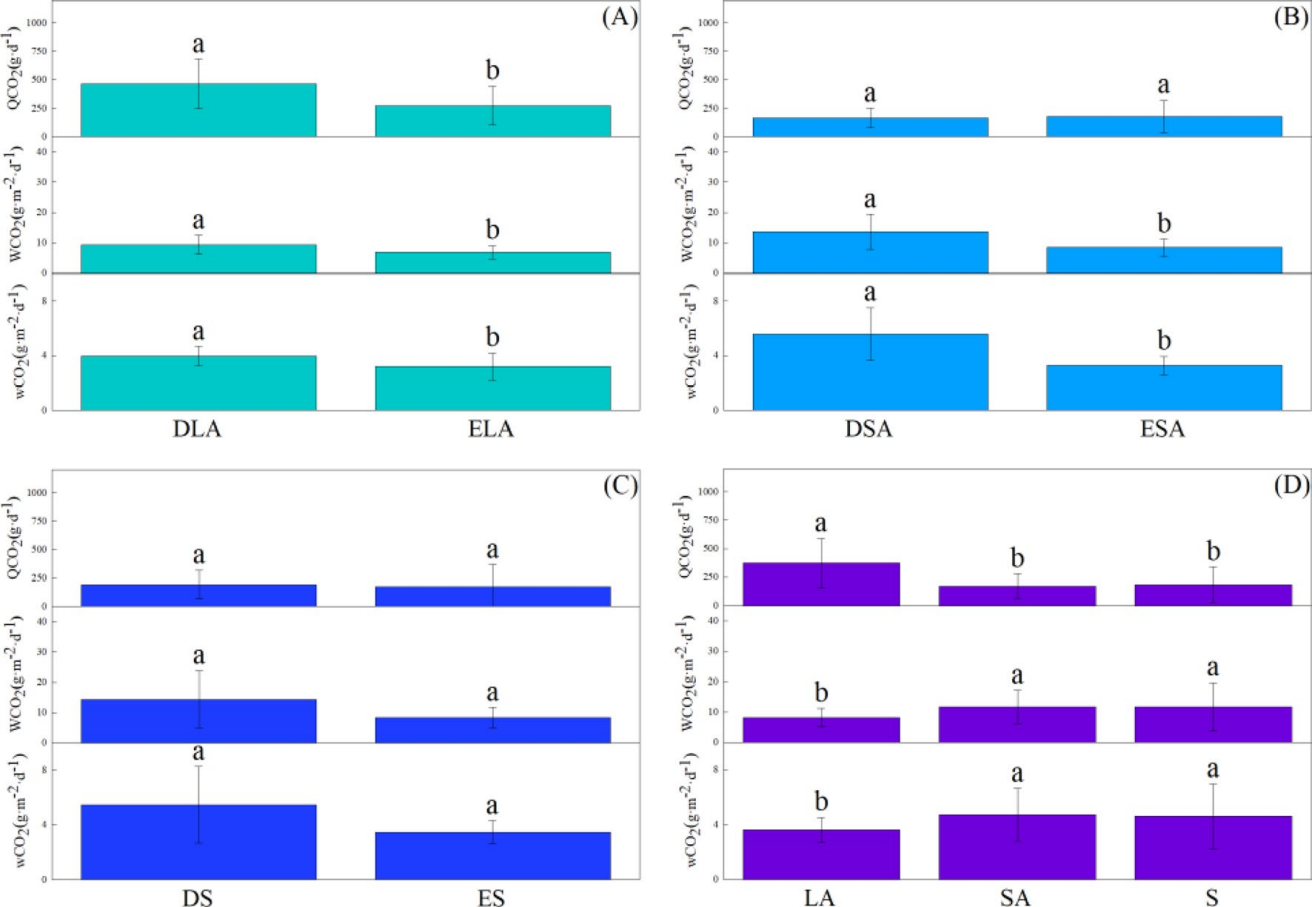
*wCO*_*2*_, *WCO*_*2*_, *QCO*_*2*_ of different plant configuration types. A, B, C, D represent the amount of carbon sequestration (*wCO*_*2*_, *WCO*_*2*_, *QCO*_*2*_) between DLA and ELA, DSA and ESA, DS and ES, LA, SA and S, respectively. The same letter in the figure indicates it has no significant difference between two tree species, or vice versa (*P* < *0.05*). The representation of DLA and ELA, DSA and ESA, DS and ES, LA, SA and S is the same as Table 2

Comparison results of *wCO*_*2*_, *WCO*_*2*_ and *QCO*_*2*_ by one-way ANOVA among tree species in each vegetation type and among six plant configuration types were presented in Fig. 1 and Fig. 2, respectively, which could concluded that variation laws of these three indexes appeared different features due to trees discrepancies of morphological and photosynthetic characteristics (Table 2, 3).

The top three species of *wCO*_*2*_, *WCO*_*2*_ and *QCO*_*2*_ were shown as *G. biloba* > *Populus* > *S. japonicum*, *G. biloba* > *A. buergerianum* > *Populus*, *P. stenoptera* > *P. acerifolia* > *Populus*, respectively among DLA, where *wCO*_*2*_ of *G. biloba* was significantly higher than that of *S. Japonicum,* and *WCO*_*2*_ of *G. biloba* was significantly greater than that of *A. buergerianum* and *Populus*, and while for *QCO*_*2*_, there was no significant difference between the three species *(P* < *0.05*). Besides, *K. paniculata* had the lowest *wCO*_2_ and *WCO*_2_, which only accounted for 54.2% and 34.8% of *G. biloba*, respectively, and *S. babylonica* had the lowest *QCO*_*2*_, which accounted for 28.6% of *P*. *stenoptera* (Fig. 1A). Among ELA, the top three species of *wCO*_2_, *WCO*_2_ and *QCO*_2_ were shown as *P. orientalis* > *S. chinensis* > *L. compactum*, *P. orientalis* > *S. chinensis* > *L. compactum*, and *C. camphora* > *S. chinensis* > *M. grandiflora*, respectively, in which *wCO*_2_ of *P. orientalis* was significantly higher than that of *L. compactum*. But for *WCO*_2_ and *QCO*_2_, it had no significant differences among the three species (*P* < *0.05*). Also, the lowest *wCO*_2_, *WCO*_2_ and *QCO*_2_ were observed both in *J. formosana* (Fig. 1B).

Figure 1(C) indicated that among DSA, the top three species of *wCO*_*2*_ and *WCO*_*2*_ were both shown as *A. persica ‘Duplex’* > *S. oblata* > *P. cerasifera ‘Atropurpure’*, and *A. persica 'Dupl*ex' was significantly higher than other tree species (*P* < *0.05*). *P. Cerasys* had the lowest *wCO*_*2*_, while *C. chinensis* had the largest *WCO*_*2*_, which accounted for 42.4% and 38.5% of *A. persista 'Duplex'*, respectively. Different from *wCO*_*2*_ and *WCO*_*2*_, the top three tree species of *QCO*_*2*_ were shown as *C chinensis* > *P. Cerasys* > *P. cerasifera 'Atropurpure'*, and the largest *QCO*_*2*_ was observed in *S. oblata*, which only accounted for 28.4% of *C. chinensis*. From Fig. 1D, among ESA, *wCO*_*2*_, *WCO*_*2*_ and *QCO*_*2*_ of *O. fragrans* were significantly higher than those of *P. serratifolia* and *V. odoratissimum*, but there was no significant difference between this two species (*P* < *0.05*).

Figure 1E indicated that among DS, both *wCO*_*2*_ and *WCO*_*2*_ were shown as *A. triloba* > *P. granatum* > *C. praecox* > *L. quihoui*, and *A. triloba* was 1.9 ~ 3.5 times and 2.2 ~ 5.2 times higher than other tree species, respectively. But *QCO*_*2*_ was shown as *C. praecox* > *A. triloba* > *P. granatum* > *L. quihoui*, and there was no significant difference between the four species (*P < 0.05*). From Fig. 1F, among ES, it showed *B. megistophylla* had the largest *wCO*_*2*_, which was 1.0 times higher than *P. glabratum* and 1.7 times higher than *B. sinica var. parvifolia*. And *P. glabratum* had the largest *WCO*_*2*_ and *QCO*_*2*_, which was 1.8 times and 2.0 times higher than *B. megistophylla*, respectively, and 7.5 times and 8.7 times higher than *B. sinica var. parvifolia*, respectively.

As shown in Fig. 2A, B and C, *wCO*_*2*_, *WCO*_*2*_ and *QCO*_*2*_ of DLA were significantly higher than those of ELA, and *wCO*_*2*_, *WCO*_*2*_ and *QCO*_*2*_ of DSA were also significantly higher than those of ESA, but it had no significant difference between DS and ES (*P* < *0.05*). On the whole, *wCO*_*2*_ and *WCO*_*2*_ were shown as SA > S > LA, while *QCO*_*2*_ was shown as LA > SA > S, and there was no obvious difference of *wCO*_*2*_, *WCO*_*2*_, *QCO*_*2*_ between SA and S (*P* < *0.05*) (Fig. 2D).

### Comprehensive carbon sequestration potential of different tree species

Cluster analysis was conducted based on *wCO*_*2*_, *WCO*_*2*_ and *QCO*_*2*_ of 32 landscape tree species. Results were shown in Table 4. It can be seen that the comprehensive carbon sequestration potential of all tree species was divided into five levels, and the top 10 tree species included of 5 LA (3 DLA, 2 ELA), 3 SA (1 DSA, 2 ESA) and 2 S (2 ES). Specifically, the descending order in terms of their comprehensive carbon sequestration potential was *Populus*, *P. stenoptera*, *P. acerifolia*, *V. odoratissimum*, *P. bungeana*, *P. granatum*, *S. oblata*, *J. formosana*, *B. sinica var. parvifolia*,* B. megistophylla.*

**Table 4:** Cluster classification and ranking of carbon sequestration potential of different plant configuration types

Plant configuration types	Tree species	Cluster grading	Total sort	Sorting by plant configuration types
DLA^1^	*Populus*	I	1	1
DLA	*P. stenoptera*	II	2	2
DLA	*P. acerifolia*	II	3	3
ELA^2^	*P. bungeana*	III	5	4
ELA	*J. formosana*	III	8	5
ELA	*P. orientalis*	III	14	6
DLA	*S. babylonica*	IV	19	7
DLA	*G. biloba*	V	20	8
ELA	*L. compactum*	V	21	9
DLA	*E. japonica*	V	22	10
DLA	*S. japonicum*	V	24	11
ELA	*Y. denudata*	V	26	12
DLA	*K. paniculata*	V	27	13
ELA	*C. camphora*	V	28	14
ELA	*S. chinensis*	V	29	15
ELA	*M. grandiflora*	V	30	16
DLA	*A. buergerianum*	V	32	17
ESA^3^	*P.serratifolia*	III	4	1
ESA	*O. fragrans*	III	6	2
DSA^4^	*S. oblata*	III	7	3
DSA	*P. Cerasifera 'Atropurpurea'*	III	11	4
DSA	*A. Persica 'Duplex'*	III	12	5
DSA	*P. Cerasys*	IV	16	6
ESA	*V. odoratissimum*	V	23	7
DSA	*C. chinensis*	V	25	8
ES^5^	*B. sinica var. parvifolia*	III	9	1
ES	*B. megistophylla*	III	10	2
ES	*L. quihoui*	III	13	3
DS^6^	*P. granatum*	IV	15	4
DS	*A. triloba*	IV	17	5
DS	*C. praecox*	IV	18	6
ES	*P. glabratum*	V	31	7

The representation of ^1^DLA, ^2^ELA, ^3^DSA, ^4^ESA, ^5^DS and ^6^ES is the same as Table 2

Based on the above cluster analysis results, we rearranged the order of all tree species in the light of LA, SA and S, respectively. It showed that the top three tree species among LA were *Populus*, *P. stenoptera*, *P. acerifolia*, respectively, and were *P.serratifolia*, *O. fragrans*, *S. oblata* among SA, and were *B. sinica var. parvifolia*, *B. megistophylla*, *L. quihoui* among S.

### RDA analysis of carbon sequestration potential of different plant configuration types

Redundancy (*RDA*) analysis method was used to analyze the correlation between photosynthetic and morphological characteristics and carbon sequestration potential (*wCO*_*2*_, *WCO*_*2*_, *QCO*_*2*_) in LA, SA and S. Results displayed that for LA, SA and S, the characteristic values of RDA on the first ordination axis were 0.7734, 0.5648, 0.769, respectively, and were 0.1564, 0.3854 and 0.1845 on the second ordination axis, respectively. Besides, the cumulative interpretation rates of the first and second axes were 92.98%, 95.02%, 95.35% for LA, SA and S, respectively, and the overall interpretation rates were 98.78%, 98.75%, 98.92%, respectively (Table 5). Monte Carlo test indicated that the first ranking axis and all ranking axes of three plant configuration types reached a significant level (*P* < *0.05*), which suggested a statistically significant result. Further analysis found that the first two ordination axes of RDA of three types can better reflect the correlation between various indicators of tree species and carbon sequestration potential, and their correlation was mainly determined by the first ordination axis.

**Table 5:** Redundancy analysis ordination results of photosynthetic and morphological indicators and carbon sequestration potential in LA, SA, and S

Item	LA^1^				SA^2^				S^3^			
	Axis1	Axis2	Axis3	Axis4	Axis1	Axis2	Axis3	Axis4	Axis1	Axis2	Axis3	Axis 4
Eigenvalues	0.7734	0.1564	0.0114	0.0515	0.5648	0.3854	0.012	0.0332	0.769	0.1845	0.0105	0.033
Explained variation (cumulative)	77.34	92.98	94.12	99.27	56.48	95.02	96.22	99.55	76.9	95.35	96.4	99.7
Pseudo-canonical correlation	0.969	0.9753	0.9815	0	0.9742	0.9908	0.9911	0	0.9797	0.9922	0.9593	0
Explained fitted variation (cumulative)	82.17	98.78	100		58.7	98.75	100		79.78	98.92	100	
Permutation test on first axes (F test)	F = 140, P = 0.002				F = 19.2, P = 0.002				F = 36.6, P = 0.002			
Permutation test on all axes (F test)	F = 72.9, P = 0.002				F = 39.7, P = 0.002				F = 32.7, P = 0.002			

The representation of ^1^LA, ^2^SA, ^3^S is the same as Table 2

**Fig. 3:**
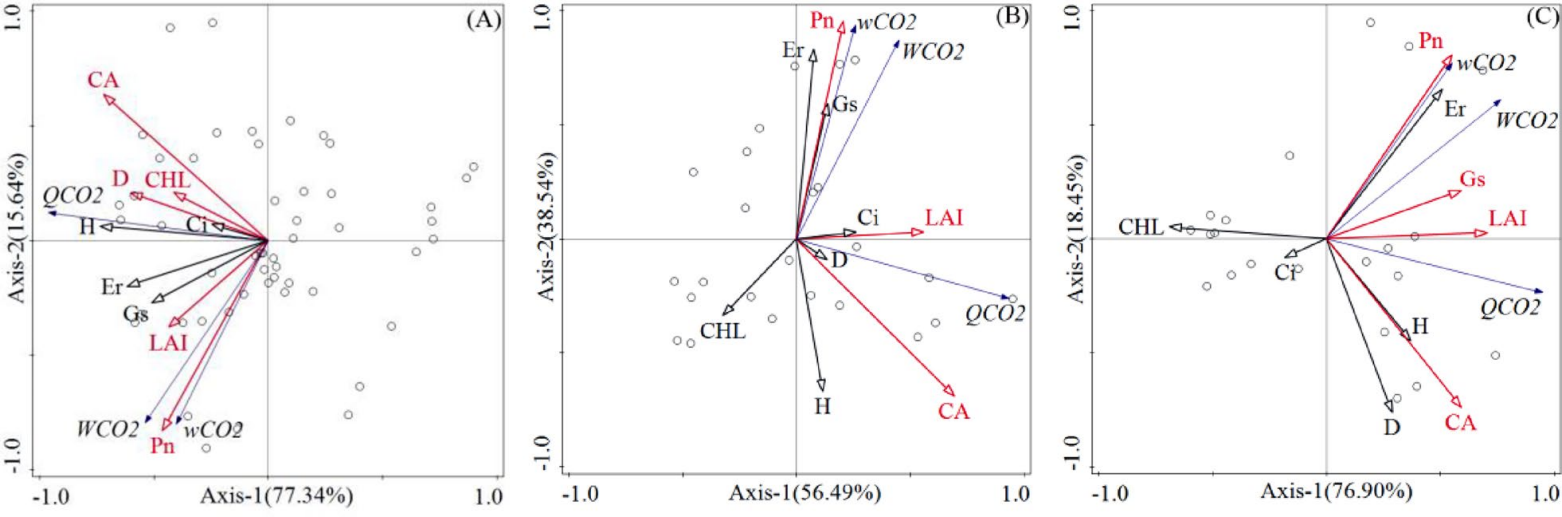
Redundancy analysis of correlation between photosynthetic and morphological indicators and trees carbon sequestration potential. A, B, C represent the redundancy analysis of correlation between photosynthetic and morphological indicators and trees carbon sequestration potential in LA (large arbors), SA (small arbors) and S (shrubs), respectively. The representation of *CA*, *LAI*, *DBH, H is the same as *Table 2*, and Pn*, *CHI*, *Ci*, *Er*, *Gs* was the same as Table3

For LA, *CA*(F = 42.4, *P* < *0.05*), *Pn* (F = 76.2, *P* < *0.05*), *LAI* (F = 25, *P* < *0.05*), *CHI*(F = 22.4, *P* < *0.05*) and *DBH* (F = 17.8, *P* < *0.05*) had a significant impact on trees comprehensive carbon sequestration potential, and the correlation was shown as *CA* > *Pn* > *LAI* > *CHI* > *DBH*, with corresponding explanatory amounts of 46.4%, 32.9%, 7.2%, 4.4% and 2.6%, respectively (Table 6). Specifically, *CA* was negatively correlated with *wCO*_*2*_ and *WCO*_*2*_, and positively correlated with *QCO*_*2*_, while other indicators were positively correlated with *wCO*_*2*_, *WCO*_*2*_ and *QCO*_*2*_ (Fig. 3A).

**Table 6:** Contribution of photosynthetic and morphological indicators to carbon sequestration potential in LA, SA, and S

LA^10^				SA^11^				S^12^			
Indicator	Explains (%)	F	P	Indicator	Explains (%)	F	P	Indicator	Explains (%)	F	P
*CA*^*1*^	46.4	42.4	0.002	*CA*	45.6	18.5	0.002	*LAI*	38.9	12.1	0.002
*Pn*^*2*^	32.9	76.2	0.002	*Pn*	41.4	66.7	0.002	*Pn*	29.1	73.3	0.002
*LAI*^*3*^	7.2	25	0.002	*LAI*	7.3	25.2	0.002	*CA*	25.2	12.7	0.002
*CHL*^*4*^	4.4	22.4	0.002	*Er*	0.8	3.1	0.086	*Gs*	1.7	5.5	0.038
*DBH*^*5*^	2.6	17.8	0.002	*CHL*	0.5	1.9	0.178	*Ci*	0.2	0.8	0.41
*Ci*^*6*^	0.3	2.1	0.132	*H*	0.4	1.6	0.178	*H*	0.4	1.3	0.306
*H*^*7*^	0.2	1.8	0.184	*Ci*	0.3	1.2	0.228	*DBH*	0.3	0.9	0.384
*Er*^*8*^	< 0.1	0.6	0.454	*DBH*	< 0.1	< 0.1	0.938	*CHL*	0.2	0.6	0.502
*Gs*^*9*^	< 0.1	< 0.1	0.934	*Gs*	< 0.1	< 0.1	0.952	*Er*	0.3	0.9	0.376

The representation of 1*CA*, 3*LAI*, 5*DBH,* 7*H is the same as *Table 2*; The representation of* *2Pn*, 4*CHI*, 6* Ci*, 8*Er* and 9*Gs* is the same as Table3; The representation of ^10^LA, ^11^SA, ^12^S is the same as Table 2

For SA, *CA* (F = 18.5, *P* < *0.05*), *Fn* (F = 66.7, *P* < *0.05*), *LAI* (F = 7.3, *P* < *0.05*) had a significant impact on trees comprehensive carbon sequestration potential, and the correlation was shown as *CA* > *Pn* > *LAI*, with corresponding interpretation amounts of 45.6%, 41.4% and 7.3%, respectively (Table 6). Specifically, *CA* was positively correlated with *QCO*_*2*_, negatively correlated with *wCO*_*2*_ and *WCO*_*2*_, and *Pn* was positively correlated with *wCO*_*2*_ and *WCO*_*2*_, negatively correlated with *QCO*_*2*_, and *LAI* was positively correlated with *wCO*_*2*_, *WCO*_*2*_ and *QCO*_*2*_ (Fig. 3B).

For S, *LAI* (F = 12.1, *P* < *0.05*), *Pn* (F = 73.3, *P* < *0.05*), *CA* (F = 12.7, *P* < *0.05*) and *Gs*(F = 5.5, *P* < *0.05*) had a significant impact on trees comprehensive carbon sequestration potential, and the correlation was shown as *LAI* > *Pn* > *CA* > *Gs*, with corresponding interpretation amounts of 38.9%, 29.1%, 25.2% and 1.7%, respectively (Table 6). Specifically, *CA* was positively correlated with *QCO*_*2*_, negatively correlated with *wCO*_*2*_ and *WCO*_*2*_, and *Pn*, *LAI* and *Gs* were positively correlated with *wCO*_*2*_, *WCO*_*2*_ and *QCO*_*2*_ (Fig. 3C).

Summarily, *CA*, *LAI*, *Pn* were the main factors which both affected the comprehensive carbon sequestration potential of LA, SA and S, even though *CA* had a more interpretation amounts among the three indicators in LA and SA, and *LAI* had a more interpretation amounts in S.

## Discussion

The 32 tree species we selected in this study all belonged to the keynote and backbone landscape tree species in Zhengzhou, of which large arbors (LA), small arbors (SA), shrubs (S) accounted for 53%, 25%, 22% for,respectively, and deciduous trees (D), evergreen trees (E) accounted for 56%, 44%, respectively. It was basically consistent with the current vegetation type of local urban green space, and represented the requirements for plant selection in the area's landscape construction.

The function of urban green space in mitigating *CO*_*2*_ has been gradually emphasized in recent years, and many relevant researches have been carried out [14, 16, 54]. In our study, we found the mean value of *Pn* for D was higher than that of E, which was consistent with the conclusion of Guo Hui's assessment on carbon fixation and oxygen release of 15 native common landscape tree species in Zhengzhou [58], but we got a very lower values. This may attribute to the different seasons in the trial investigation, because photosynthetic characteristics of plants exhibited different features influenced by different seasonal environments [59–61]. However, some studies observed a completely contrary results [62, 63], since E may grow better in subtropical monsoon climate regions (like Wuhan, Hangzhou) compared with warm temperate continental monsoon climate regions (like Zhengzhou). Also, we found *wCO*_*2*_ of S was larger in contrast with LA and it exhibited a similar characteristics of plants in some southern inland cities of China [62, 64], while contrary to eastern coastal cities of China [63]. Complex reasons may cause this consequences, such as specific selected tree species, different regional environments as well as distinct study periods [30, 50]. Besides, the little variance *LAI* of different types in our study made a comparatively similar regularity between *wCO*_*2*_ and *WCO*_*2*_, while the large disparity of crown width made them an obvious discrepancy with *QCO*_*2*_, and caused *QCO*_*2*_ of LA and SA exceeded that of S. Therefore, arbor trees played an important ecological role in landscaping construction system. Shrubs had a strong carbon sequestration potential, but their small morphological characteristics lead to less total carbon sequestration. Therefore, *LAI* and crown width can be increased through dense planting methods such as group planting and cluster planting, so as to increase their total carbon fixation [58].

Carbon sequestration potential also varied greatly among tree species in a same plant configuration type. For examples, our results showed that *A. triloba*(9.8 gm^−2^ d^−1^), *A. persica ‘Duplex’*(8.7 gm^−2^ d^−1^) and *P. cerasifera 'Atropurpurea'*(5.6 gm^−2^ d^−1^) had a higher *wCO*_*2*_, and *P. stenoptera*(4.0 gm^−2^ d^−1^), *S. babylonica*(3.8 gm^−2^ d^−1^), *P. acerifolia*(3.5 gm^−2^ d^−1^), *C. camphora*(2.2 gm^−2^ d^−1^) had a lower *wCO*_*2*_. However,,*wCO2* of *C. camphora* in northern Zhejiang Province was large (11.374 gm^−2^ d^−1^) while *P. cerasifera ‘Atropurpurea’* was small (2.178 gm^−2^ d^−1^) [53]. Except that,, *wCO*_*2*_ of *A. triloba* (6.79 gm^−2^ d^−1^) in Shenyang was smaller compared with other six common garden plants, such as *Q. mongolica* and *P. alba* × *P.beriliensis* [65]. And *wCO*_*2*_ of *C. camphora* (6-10 gm^−2^ d^−1^) in Shanghai was lower than that of *S. babylonica* (> 12 gm^−2^ d^−1^) [66]. We also observed that *WCO*_*2*_ of *M. grandiflora* was 6.1 g.m^−2^.d^−1^ in Zhengzhou, but it was 46.49 g.m^−2^.d^−1^ in Shanghai’s garden plants community [32] and 174.03 gm^−2^ d^−1^ in Kunming’s road greening trees [29]. This suggested that *WCO*_*2*_ seemed to present an increasing trend from north to south in China. In addition, several arbor trees, such as *P. stenoptera*, *P. acerifolia* and *C. camphora*, showed high in *QCO*_*2*_ (exceeding 400 g d^−1^) in our study, which was similar to the conclusions of Guo Hui and Zhao Yanling [32, 58].

*wCO*_*2*_ reflected the strength of plants carbon sequestration potential through their leaf photosynthesis, and for that, it was directly applied for evaluating the carbon sequestration potential of different tree species [53, 66]. However, it seemed that *QCO*_*2*_ could better represent the comprehensive level of carbon sequestration of individual plants and ecological function of urban green space, which also was considered a better scientific evaluation standard [32, 58, 62, 65]. However, Zhang Na advised taking the ornamental value of trees and its *QCO*_*2*_ into account at the same time [67]. Besides that, some scholars adopted other statistical analysis methods to make an overall evaluation from plant photosynthetic, physiological, or transpiration characteristics [53, 68]. Nevertheless, we believed that *wCO*_*2*_, *WCO*_*2*_ and *QCO*_*2*_ respectively represented three different perspectives of plants carbon sequestration potential, and appropriate indicators should be selected under various conditions for analysis. Specifically, tree species with high *wCO*_*2*_ can be given priority on the condition of thinking about plants photosynthetic carbon fixation capacity, and tree species with high *WCO*_*2*_ could be considered in the case of limited urban green space, and tree species with high *QCO*_*2*_ should be suggested when the number of seedlings was determined. However, we preferred that tree species with both higher *wCO*_*2*_, *WCO*_*2*_ and *QCO*_*2*_ were recommended, such as *Populus*, *P. stenoptera*, *P. acifolia* in LA, *P. serratifolia*, *O. fragrans*, *S. oblata* in SA, and *B. sinica var. parvifolia*, *B. megistophylla*, *L. quihoui* in S.

Previous studied showed that *Pn* and *Er* were extremely significant correlated with *wCO*_*2*_ [68, 69], and *LAI* and *DBH* were extremely significant correlated with *WCO*_*2*_, while *CD*, *DBH*, *H* and *LAI* were extremely significant correlated with *QCO*_*2*_, respectively [32]. This was very different from the conclusion of our study, because we paid more attention to the factors that jointly affected *wCO*_*2*_, *WCO*_*2*_ and *QCO*_*2*_, and found that *CA*, *LAI* and *Pn* could significantly affect the comprehensive carbon fixation potential of landscape tree species both in LA, SA and S.

Briefly, arbor trees should be chosen as the main greening plants, while shrubs and herbs as the auxiliary plants in terms of the allocation mode of urban greening vegetation, and higher carbon sequestration potential tree species in different plant configuration types should also be selected. Meanwhile, we’d better consider an appropriate proportion of deciduous and evergreen tree species to build a multi-layer community structure with high biodiversity, so that it can reasonably use water, light, temperature, space and other resources, to improve the ecological and economic comprehensive benefits of the entire urban green space. In this study, we didn’t access the carbon sequestration potential of herbs, so it will be considered in the future research. Moreover, the work we did lean towards a fundamental research, and our analysis about the assessment of carbon sequestration potential of trees was mainly based on trees photosynthetic capacity. However, trees carbon sequestration potential were also related to their biomass, age, growth environment conditions, management level and so on, so we will consider these factors comprehensively by combing practical applications and try to provide a more appropriate method for city policymakers. Besides that, we provided a ranking list of 32 common landscape trees based on the condition of our study areas, which may give a basis for the selection of landscape tree species in Zhengzhou or similar areas.

## Conclusion

It had a different carbon sequestration potential among the selected 32 common landscape tree species by comparing their *wCO*_*2*_, *WCO*_*2*_ and *QCO*_*2*_. Comprehensive evaluation results of *wCO*_*2*_, *WCO*_*2*_ and *QCO*_*2*_ by cluster analysis showed that the top 10 tree species of high carbon sequestration potential include 3 DLA (Deciduous large arbor), 2 ELA (Evergreen large arbor), 1 DSA (Evergreen small arbor), 2 ESA (Evergreen small arbor) and 2 ES (Evergreen shrubs). Considering that hierarchical landscape spaces are generally formed in urban landscaping, we recommend using *Populus*, *P. stenoptera*, *P. acerifolia* among LA (Large arbors), and *P.serratifolia*, *O. fragrans*, *S. oblata* among SA (Small arbor), and *B. sinica var. parvifolia*, *B. megistophylla*, *L. quihoui* among S (Shrub). *Pn*, *CA* and *LAI* were the main factors which affected the comprehensive carbon sequestration potential of different plant configuration types.

## Data Availability

The dataset supporting the conclusions of this article is included within the article.
